# Carbonyl­bis­{6,6′-[(3,3′-di-*tert*-butyl-5,5′-dimeth­oxy-1,1′-biphenyl-2,2′-di­yl)bis(oxy)]bis­(dibenzo[*d*,*f*][1,3,2]-dioxaphosphepine)}hydridorhodium(I) toluene-*d*_8_ 2.25-solvate

**DOI:** 10.1107/S2414314623000834

**Published:** 2023-02-14

**Authors:** Benedict N. Leidecker, Anke Spannenberg, Robert Franke, Armin Börner, Christoph Kubis

**Affiliations:** a Leibniz-Institut für Katalyse e. V., Albert-Einstein-Str. 29a, 18059 Rostock, Germany; b Evonik Operations GmbH, Paul-Baumann-Str. 1, 45772 Marl, Germany; c Lehrstuhl für Theoretische Chemie, Ruhr-Universität Bochum, 44780 Bochum, Germany; Benemérita Universidad Autónoma de Puebla, México

**Keywords:** crystal structure, bis­phosphite, Biphephos, rhodium, hydride complex

## Abstract

The crystal-structure determination of the title compound, [RhH(C_46_H_44_O_8_P_2_)_2_(CO)]·2.25C_7_D_8_, is reported. The bis­phosphite ligand, C_46_H_44_O_8_P_2_, is well known as Biphephos. One specific characteristic of this hydrido rhodium(I) monocarbonyl complex is that one bis­phosphite ligand is coordinated in the expected bidentate mode and the other is coordinated in a monodentate mode. Thus, one phosphite moiety remains non-coordinating. All three coordinating phospho­rus atoms occupy the equatorial positions in the trigonal–bipyramidal environment around the rhodium atom. The crystals of the hydrido rhodium(I) monocarbonyl complex contains deuterated solvent mol­ecules (toluene-*d*_8_). Most of them are included in the model, but the contributions of about 0.84 toluene per unit cell were removed from the diffraction data, using the SQUEEZE procedure in *PLATON* [Spek (2015 ▸). *Acta Cryst.* C**71**, 9–18].

## Abstract 2

In the title hydrido rhodium(I) monocarbonyl complex, one bis­phosphite ligand coordinates in the expected bidentate mode, whereas the other coordinates to the metal in a monodentate mode.

## Graphical abstract

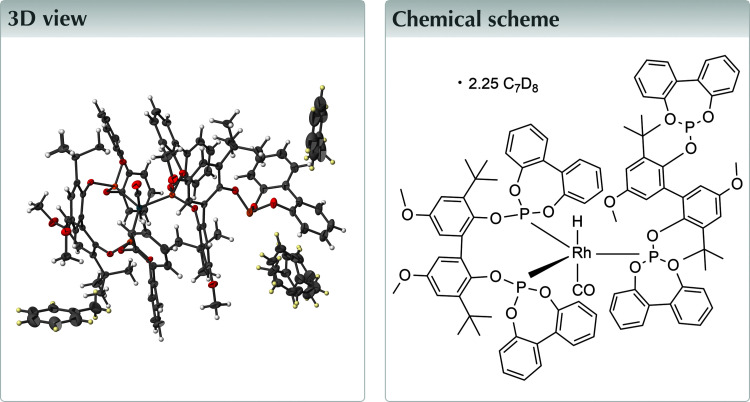

## Structure description

**Figure 1:**
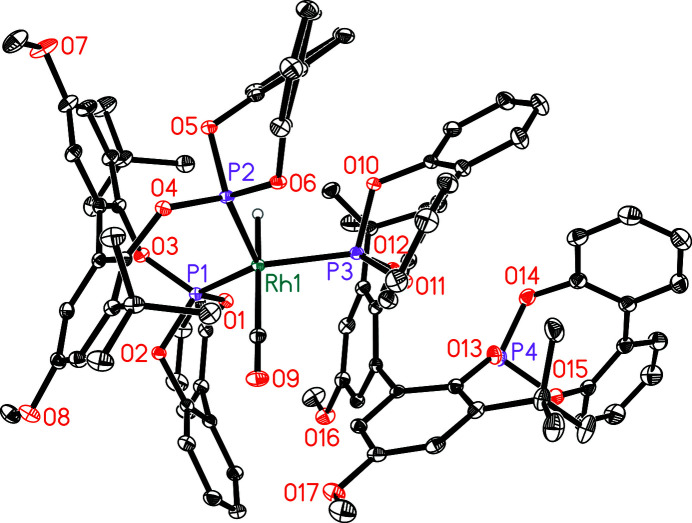
Mol­ecular structure of the title compound with displacement ellipsoids drawn at 30% probability level. Co-crystallized solvent and C–bound hydrogen atoms are omitted for clarity.

**Figure 2:**
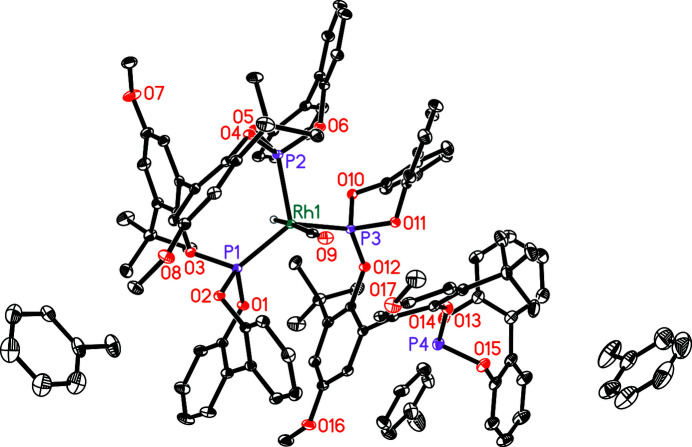
Mol­ecular structure of the title compound with displacement ellipsoids drawn at 30% probability level. Deuterium and C–bound hydrogen atoms are omitted for clarity. Disordered solvent mol­ecules are shown in only one orientation.

The crystal structure of the bis­phosphite ligand Biphephos has recently been studied (Leidecker *et al.*, 2019 ▸). Biphephos is applied as a co-catalyst for the formation of the respective bis­phosphite-modified hydrido rhodium(I) dicarbonyl complex [RhH(CO)_2_(Biphephos)] as catalyst for highly *n*-regioselective alkene hydro­formyl­ation (Börner & Franke, 2016 ▸; Moasser *et al.*, 1995 ▸). This rhodium dicarbonyl complex converts in the presence of an additional equivalent of free Biphephos and in the absence of carbon monoxide into a monocarbonyl complex, during which the carbonyl ligand in the equatorial position is substituted by one phosphite moiety. The coordination environment around the rhodium(I) atom can be described as distorted trigonal–bipyramidal with three phospho­rus atoms coordinating in the equatorial positions (Fig. 1 ▸). The occupation of the equatorial positions by the phospho­rus atoms of the chelating Biphephos ligand is known from spectroscopic studies in solution (Moasser *et al.*, 1995 ▸). The preference for the coordination at the equatorial position of the third phospho­rus atom belonging to the second ligand is caused by steric properties (Selent *et al.*, 2013 ▸; van der Veen *et al.*, 1998 ▸). The distances Rh1—P1 = 2.2325 (4) Å, Rh1—P2 = 2.2572 (4) Å and Rh1—P3 = 2.2600 (4) Å are comparable to those in complexes of the type [RhH(CO)_2_(P∩P)], with bis­phosphites as a ligand (van Rooy *et al.*, 1996 ▸; Selent *et al.*, 2012 ▸; Mormul *et al.*, 2015 ▸). The angle P1—Rh1—P2 = 118.79 (2)° is related to the chelating coordination mode of one ligand. The other P—Rh—P angles with P2—Rh1—P3 = 106.08 (2)° and P1—Rh1—P3 = 128.40 (2)° differ significantly from the ideal trigonal–bipyramidal geometry. The hydrido ligand and the carbonyl ligand coordinate at the axial positions. The crystals of the hydrido rhodium(I) monocarbonyl complex contain three sites including toluene-*d*_8_ solvent mol­ecules, two of which are partly occupied and disordered (Fig. 2 ▸). Additionally, the contributions of heavily disordered toluene-*d*_8_ (0.84 mol­ecules per unit cell) were removed from the diffraction data using the SQUEEZE procedure in *PLATON* (Spek, 2015 ▸).

## Synthesis and crystallization

The bis­phosphite Biphephos was provided by Evonik Operations GmbH (OxoPhos® 17). [Rh(acac)(CO)_2_] (25.8 mg, 0.15 mmol) and Biphephos (157.36 mg, 0.20 mmol) were dissolved in 5 ml of toluene-*d*_8_, transferred into a stainless-steel reactor (Swagelock®) and treated with synthesis gas (20 bar) at 50°C for 2 h. After the reaction, the pressure was reduced to atmospheric pressure of the synthesis gas. Afterwards the sample was purged 12 times with pure hydrogen and the gas atmosphere completely exchanged and then cooled down to room temperature. During slow evaporation of toluene-*d*_8_ at room temperature, fine colourless crystals were obtained and measured *via* single-crystal X-ray diffraction. The catalyst pre-formation starting from [Rh(acac)(CO)_2_] as a precursor in the presence of a bis­phosphite ligand under *in situ* conditions at elevated pressures of synthesis gas (CO/H_2_) is an established procedure (Börner & Franke, 2016 ▸). Toluene-*d*_8_ was chosen because the highly concentrated catalyst solution was also used for further NMR-spectroscopic measurements. Crystallization of the carbonyl hydrido Rh^I^ complex might also be possible from other solvents such as non-deuterated benzene or toluene.

## Refinement

Crystal data, data collection and structure refinement details are summarized in Table 1 ▸. The hydride (H1) could be found from the difference-Fourier map and was refined with free coordinates. The crystals of the hydrido rhodium(I) monocarbonyl complex contain solvent (toluene-*d*_8_). The disordered solvent mol­ecules were refined with the benzene rings constrained to have an idealized geometry (flat hexa­gon with C—C bond lengths of 1.39 Å). Moreover, disordered solvent mol­ecules placed on the same site were restrained to have similar *U*_ij_ parameters, with standard deviation of 0.04 Å^2^ (Sheldrick, 2015*b* ▸). The contributions of some additional disordered solvent were removed from the diffraction data using the SQUEEZE procedure in *PLATON* (Spek, 2015 ▸). SQUEEZE estimated the electron counts in the void of 197 Å^3^ volume to be 42, which corresponds to 0.84 disordered toluene mol­ecules per triclinic unit cell.

## Supplementary Material

Crystal structure: contains datablock(s) I, global. DOI: 10.1107/S2414314623000834/bh4071sup1.cif

Structure factors: contains datablock(s) I. DOI: 10.1107/S2414314623000834/bh4071Isup2.hkl

CCDC reference: 2238871

Additional supporting information:  crystallographic information; 3D view; checkCIF report

## Figures and Tables

**Table 1:** Experimental details

Crystal data	
Chemical formula	[RhH(C_46_H_44_O_8_P_2_)_2_(CO)]·2.25C_7_D_8_
*M*_r_	1930.84
Crystal system, space group	Triclinic, *P* 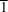
Temperature (K)	150
*a*, *b*, *c* (Å)	13.5471 (10), 18.9745 (15), 19.8784 (16)
α, β, γ (°)	77.0694 (17), 82.7785 (17), 77.2409 (17)
*V* (Å^3^)	4841.5 (7)
*Z*	2
Radiation type	Mo *K*α
μ (mm^−1^)	0.31
Crystal size (mm)	0.43 × 0.28 × 0.22

Data collection	
Diffractometer	Bruker APEXII CCD
Absorption correction	Multi-scan (*SADABS*; Bruker, 2016 ▸)
*T*_min_, *T*_max_	0.88, 0.94
No. of measured, independent and observed [*I* > 2σ(*I*)] reflections	143429, 23351, 20576
*R*_int_	0.030
(sin θ/λ)_max_ (Å^−1^)	0.661

Refinement	
*R*[*F* ^2^ > 2σ(*F* ^2^)], *wR*(*F* ^2^), *S*	0.031, 0.087, 1.04
No. of reflections	23351
No. of parameters	1261
No. of restraints	258
H-atom treatment	H atoms treated by a mixture of independent and constrained refinement
Δρ_max_, Δρ_min_ (e Å^−3^)	0.81, −0.79

Computer programs: *APEX3* and *SAINT* (Bruker, 2019 ▸), *SHELXT2014* (Sheldrick, 2015*a* ▸), *SHELXL2018* (Sheldrick, 2015*b* ▸), *XP* in *SHELXTL* (Sheldrick, 2008 ▸) and *publCIF* (Westrip, 2010 ▸).

## full crystallographic data

### Crystal data

**Table:**

[RhH(C_46_H_44_O_8_P_2_)_2_(CO)]·2.25C_7_D_8_	*Z* = 2
*M**_r_* = 1930.84	*F*(000) = 2001
Triclinic, *P*1	*D*_x_ = 1.324 Mg m^−^^3^
*a* = 13.5471 (10) Å	Mo *K*α radiation, λ = 0.71073 Å
*b* = 18.9745 (15) Å	Cell parameters from 9835 reflections
*c* = 19.8784 (16) Å	θ = 2.3–30.6°
α = 77.0694 (17)°	µ = 0.31 mm^−^^1^
β = 82.7785 (17)°	*T* = 150 K
γ = 77.2409 (17)°	Prism, colourless
*V* = 4841.5 (7) Å^3^	0.43 × 0.28 × 0.22 mm

### Data collection

**Table:**

Bruker APEXII CCD diffractometer	23351 independent reflections
Radiation source: fine-focus sealed tube	20576 reflections with *I* > 2σ(*I*)
Detector resolution: 8.3333 pixels mm^-1^	*R*_int_ = 0.030
φ and ω scans	θ_max_ = 28.0°, θ_min_ = 1.7°
Absorption correction: multi-scan (SADABS; Bruker, 2016)	*h* = −17→17
*T*_min_ = 0.88, *T*_max_ = 0.94	*k* = −25→25
143429 measured reflections	*l* = −26→26

### Refinement

**Table:**

Refinement on *F*^2^	Primary atom site location: dual
Least-squares matrix: full	Secondary atom site location: difference Fourier map
*R*[*F*^2^ > 2σ(*F*^2^)] = 0.031	Hydrogen site location: mixed
*wR*(*F*^2^) = 0.087	H atoms treated by a mixture of independent and constrained refinement
*S* = 1.04	*w* = 1/[σ^2^(*F*_o_^2^) + (0.042*P*)^2^ + 3.4299*P*] where *P* = (*F*_o_^2^ + 2*F*_c_^2^)/3
23351 reflections	(Δ/σ)_max_ = 0.005
1261 parameters	Δρ_max_ = 0.81 e Å^−^^3^
258 restraints	Δρ_min_ = −0.79 e Å^−^^3^

### Fractional atomic coordinates and isotropic or equivalent isotropic displacement parameters (Å^2^)

**Table:**

	*x*	*y*	*z*	*U*_iso_*/*U*_eq_	Occ. (<1)
C1	0.42212 (12)	0.25587 (9)	0.63980 (8)	0.0195 (3)	
C2	0.32459 (13)	0.24708 (10)	0.66485 (9)	0.0262 (3)	
H2	0.308763	0.231954	0.713155	0.031*	
C3	0.25002 (14)	0.26048 (12)	0.61908 (10)	0.0342 (4)	
H3	0.183389	0.253338	0.635867	0.041*	
C4	0.27291 (14)	0.28421 (13)	0.54907 (10)	0.0376 (5)	
H4	0.221752	0.293947	0.517751	0.045*	
C5	0.36990 (14)	0.29378 (11)	0.52460 (9)	0.0301 (4)	
H5	0.384595	0.310262	0.476349	0.036*	
C6	0.44721 (12)	0.27975 (9)	0.56924 (8)	0.0215 (3)	
C7	0.55070 (12)	0.28787 (9)	0.54017 (8)	0.0200 (3)	
C8	0.56699 (14)	0.34519 (9)	0.48448 (8)	0.0254 (3)	
H8	0.511279	0.383168	0.468993	0.030*	
C9	0.66276 (15)	0.34736 (10)	0.45171 (9)	0.0285 (4)	
H9	0.672561	0.386993	0.414420	0.034*	
C10	0.74467 (14)	0.29182 (10)	0.47313 (9)	0.0274 (4)	
H10	0.810079	0.292541	0.449590	0.033*	
C11	0.73085 (13)	0.23522 (10)	0.52898 (8)	0.0229 (3)	
H11	0.786702	0.197205	0.544126	0.027*	
C12	0.63514 (12)	0.23462 (8)	0.56238 (8)	0.0182 (3)	
C13	0.59038 (12)	0.05302 (8)	0.77726 (8)	0.0179 (3)	
C14	0.51693 (12)	0.02764 (9)	0.82873 (9)	0.0220 (3)	
C15	0.55128 (13)	−0.03125 (10)	0.88114 (9)	0.0286 (4)	
H15	0.503871	−0.048132	0.917252	0.034*	
C16	0.65253 (13)	−0.06635 (10)	0.88262 (9)	0.0268 (4)	
C17	0.72217 (12)	−0.04354 (9)	0.83031 (9)	0.0220 (3)	
H17	0.791109	−0.068632	0.830406	0.026*	
C18	0.69157 (12)	0.01671 (8)	0.77682 (8)	0.0180 (3)	
C19	0.77139 (11)	0.03281 (8)	0.71980 (8)	0.0171 (3)	
C20	0.75729 (12)	0.02787 (8)	0.65281 (8)	0.0195 (3)	
H20	0.693878	0.021300	0.642210	0.023*	
C21	0.83651 (12)	0.03264 (9)	0.60206 (8)	0.0210 (3)	
C22	0.93152 (12)	0.03660 (9)	0.61895 (8)	0.0218 (3)	
H22	0.986484	0.035555	0.584192	0.026*	
C23	0.94881 (12)	0.04204 (9)	0.68493 (8)	0.0187 (3)	
C24	0.86465 (11)	0.04375 (8)	0.73404 (8)	0.0170 (3)	
C25	0.40242 (12)	0.05992 (10)	0.82745 (9)	0.0260 (3)	
C26	0.34023 (15)	0.01518 (13)	0.88465 (12)	0.0437 (5)	
H26A	0.353568	−0.036112	0.878863	0.066*	
H26B	0.267748	0.036479	0.881478	0.066*	
H26C	0.359760	0.016677	0.930063	0.066*	
C27	0.37977 (14)	0.13918 (10)	0.83949 (10)	0.0297 (4)	
H27A	0.397047	0.138879	0.885981	0.045*	
H27B	0.307457	0.160421	0.835253	0.045*	
H27C	0.420336	0.168886	0.804922	0.045*	
C28	0.36607 (14)	0.05719 (11)	0.75823 (11)	0.0324 (4)	
H28A	0.401237	0.087404	0.720327	0.049*	
H28B	0.292704	0.076368	0.758271	0.049*	
H28C	0.381036	0.006049	0.751853	0.049*	
C29	0.77303 (15)	−0.16684 (11)	0.93774 (12)	0.0399 (5)	
H29A	0.787523	−0.188646	0.896416	0.060*	
H29B	0.778563	−0.206213	0.979281	0.060*	
H29C	0.821925	−0.135584	0.937322	0.060*	
C30	0.73383 (14)	0.02611 (11)	0.51633 (9)	0.0299 (4)	
H30A	0.716145	−0.020522	0.541910	0.045*	
H30B	0.736870	0.027838	0.466486	0.045*	
H30C	0.682196	0.067450	0.528282	0.045*	
C31	1.05574 (12)	0.04355 (9)	0.70251 (8)	0.0217 (3)	
C32	1.06044 (13)	0.12047 (10)	0.71223 (10)	0.0268 (4)	
H32A	1.127850	0.119950	0.725598	0.040*	
H32B	1.008792	0.134563	0.748594	0.040*	
H32C	1.047704	0.156162	0.668712	0.040*	
C33	1.13607 (13)	0.02420 (12)	0.64446 (9)	0.0312 (4)	
H33A	1.203608	0.022885	0.658085	0.047*	
H33B	1.122918	0.061583	0.602033	0.047*	
H33C	1.133043	−0.024244	0.636133	0.047*	
C34	1.08489 (13)	−0.01414 (10)	0.76818 (9)	0.0279 (4)	
H34A	1.080646	−0.063045	0.761656	0.042*	
H34B	1.038183	−0.001826	0.807581	0.042*	
H34C	1.154439	−0.014381	0.777220	0.042*	
C35	0.70504 (12)	0.12252 (9)	0.94496 (8)	0.0189 (3)	
C36	0.59996 (13)	0.13809 (9)	0.95051 (8)	0.0238 (3)	
H36	0.563263	0.120983	0.921847	0.029*	
C37	0.54907 (14)	0.17908 (10)	0.99859 (9)	0.0291 (4)	
H37	0.476961	0.190741	1.002691	0.035*	
C38	0.60332 (14)	0.20296 (10)	1.04056 (9)	0.0297 (4)	
H38	0.568328	0.231872	1.072800	0.036*	
C39	0.70817 (14)	0.18500 (10)	1.03584 (8)	0.0261 (3)	
H39	0.744436	0.200564	1.065900	0.031*	
C40	0.76168 (12)	0.14432 (9)	0.98758 (8)	0.0203 (3)	
C41	0.87337 (12)	0.12243 (9)	0.98350 (8)	0.0199 (3)	
C42	0.92347 (14)	0.09598 (10)	1.04425 (8)	0.0253 (3)	
H42	0.885115	0.094826	1.087774	0.030*	
C43	1.02729 (14)	0.07153 (10)	1.04241 (9)	0.0286 (4)	
H43	1.059743	0.053097	1.084296	0.034*	
C44	1.08399 (13)	0.07397 (10)	0.97928 (9)	0.0284 (4)	
H44	1.155508	0.057008	0.977852	0.034*	
C45	1.03679 (12)	0.10109 (9)	0.91807 (9)	0.0234 (3)	
H45	1.075807	0.103600	0.874725	0.028*	
C46	0.93244 (12)	0.12444 (9)	0.92069 (8)	0.0188 (3)	
C47	0.81351 (12)	0.23393 (8)	0.68053 (8)	0.0192 (3)	
C48	0.70024 (13)	0.35017 (9)	0.91966 (8)	0.0214 (3)	
C49	0.61875 (14)	0.37551 (10)	0.96322 (9)	0.0259 (3)	
H49	0.553522	0.366098	0.960404	0.031*	
C50	0.63297 (16)	0.41482 (11)	1.01110 (10)	0.0336 (4)	
H50	0.577531	0.432170	1.041466	0.040*	
C51	0.72792 (17)	0.42863 (12)	1.01447 (10)	0.0382 (5)	
H51	0.737729	0.455574	1.047183	0.046*	
C52	0.80860 (16)	0.40344 (11)	0.97050 (10)	0.0339 (4)	
H52	0.873341	0.413826	0.973041	0.041*	
C53	0.79699 (13)	0.36280 (9)	0.92213 (8)	0.0235 (3)	
C54	0.88568 (13)	0.33399 (9)	0.87779 (8)	0.0223 (3)	
C55	0.98111 (14)	0.30683 (11)	0.90344 (10)	0.0296 (4)	
H55	0.988165	0.304236	0.950933	0.036*	
C56	1.06532 (14)	0.28370 (11)	0.86118 (11)	0.0334 (4)	
H56	1.129470	0.265195	0.879742	0.040*	
C57	1.05660 (14)	0.28738 (11)	0.79183 (10)	0.0313 (4)	
H57	1.114857	0.272016	0.762691	0.038*	
C58	0.96267 (13)	0.31353 (10)	0.76485 (9)	0.0256 (3)	
H58	0.956162	0.316056	0.717294	0.031*	
C59	0.87887 (12)	0.33581 (9)	0.80783 (8)	0.0207 (3)	
C60	0.54080 (12)	0.41447 (8)	0.72704 (8)	0.0182 (3)	
C61	0.43708 (12)	0.41405 (9)	0.74140 (8)	0.0216 (3)	
C62	0.37798 (13)	0.43351 (9)	0.68472 (9)	0.0246 (3)	
H62	0.307199	0.434093	0.692641	0.029*	
C63	0.41949 (13)	0.45192 (9)	0.61750 (9)	0.0231 (3)	
C64	0.52104 (12)	0.45603 (9)	0.60494 (8)	0.0207 (3)	
H64	0.549071	0.470317	0.558895	0.025*	
C65	0.58180 (12)	0.43918 (8)	0.65986 (8)	0.0179 (3)	
C66	0.68838 (12)	0.45020 (8)	0.64356 (8)	0.0181 (3)	
C67	0.75044 (12)	0.41007 (9)	0.59845 (8)	0.0204 (3)	
H67	0.724431	0.377046	0.579173	0.024*	
C68	0.84993 (13)	0.41801 (9)	0.58152 (8)	0.0230 (3)	
C69	0.88908 (13)	0.46384 (10)	0.61169 (9)	0.0250 (3)	
H69	0.958743	0.466546	0.601667	0.030*	
C70	0.82906 (13)	0.50622 (9)	0.65647 (9)	0.0240 (3)	
C71	0.72668 (12)	0.50085 (9)	0.66933 (8)	0.0206 (3)	
C72	0.38586 (13)	0.39671 (10)	0.81529 (9)	0.0266 (4)	
C73	0.42883 (15)	0.31787 (10)	0.85263 (10)	0.0314 (4)	
H73A	0.499998	0.313809	0.860581	0.047*	
H73B	0.424437	0.282978	0.823994	0.047*	
H73C	0.389515	0.306474	0.897145	0.047*	
C74	0.27127 (15)	0.40266 (15)	0.81441 (12)	0.0474 (6)	
H74A	0.240722	0.392414	0.862048	0.071*	
H74B	0.259294	0.366778	0.789289	0.071*	
H74C	0.240548	0.452582	0.791326	0.071*	
C75	0.40062 (16)	0.45228 (10)	0.85638 (10)	0.0335 (4)	
H75A	0.370478	0.439930	0.903911	0.050*	
H75B	0.367508	0.502030	0.834459	0.050*	
H75C	0.473341	0.450477	0.857118	0.050*	
C76	0.25979 (15)	0.46869 (13)	0.57090 (12)	0.0414 (5)	
H76A	0.249057	0.420916	0.598562	0.062*	
H76B	0.231241	0.477393	0.526155	0.062*	
H76C	0.226127	0.507946	0.595569	0.062*	
C77	1.01065 (15)	0.36759 (13)	0.53015 (12)	0.0409 (5)	
H77A	1.032025	0.414601	0.510721	0.061*	
H77B	1.040316	0.332149	0.500469	0.061*	
H77C	1.033818	0.348279	0.576720	0.061*	
C78	0.87709 (14)	0.55480 (10)	0.69058 (11)	0.0319 (4)	
C79	0.83163 (18)	0.63643 (11)	0.66452 (14)	0.0458 (5)	
H79A	0.865554	0.667007	0.683988	0.069*	
H79B	0.758889	0.646113	0.679041	0.069*	
H79C	0.841367	0.648399	0.613868	0.069*	
C80	0.99240 (16)	0.54428 (13)	0.67246 (13)	0.0443 (5)	
H80A	1.006194	0.559798	0.622344	0.066*	
H80B	1.023801	0.492144	0.687024	0.066*	
H80C	1.020707	0.574199	0.696466	0.066*	
C81	0.86080 (17)	0.53374 (13)	0.76978 (11)	0.0416 (5)	
H81A	0.896973	0.483054	0.785786	0.062*	
H81B	0.788110	0.537268	0.783544	0.062*	
H81C	0.886922	0.567457	0.790575	0.062*	
C82	0.56709 (14)	0.62625 (10)	0.82060 (9)	0.0277 (4)	
C83	0.62177 (16)	0.58119 (11)	0.87378 (10)	0.0346 (4)	
H83	0.628460	0.529143	0.881850	0.042*	
C84	0.66649 (17)	0.61277 (13)	0.91499 (11)	0.0421 (5)	
H84	0.703504	0.582529	0.952145	0.051*	
C85	0.65738 (18)	0.68851 (13)	0.90215 (12)	0.0431 (5)	
H85	0.688397	0.710069	0.930584	0.052*	
C86	0.60366 (16)	0.73313 (11)	0.84842 (11)	0.0363 (4)	
H86	0.598933	0.785022	0.839838	0.044*	
C87	0.55628 (14)	0.70283 (10)	0.80662 (9)	0.0288 (4)	
C88	0.49204 (15)	0.74914 (10)	0.75185 (10)	0.0295 (4)	
C89	0.42100 (18)	0.81161 (11)	0.76359 (11)	0.0398 (5)	
H89	0.417469	0.827126	0.806225	0.048*	
C90	0.35577 (19)	0.85117 (12)	0.71406 (12)	0.0473 (6)	
H90	0.307318	0.893224	0.723052	0.057*	
C91	0.36077 (18)	0.82976 (12)	0.65144 (12)	0.0450 (5)	
H91	0.314696	0.856348	0.617945	0.054*	
C92	0.43314 (17)	0.76938 (11)	0.63753 (11)	0.0367 (4)	
H92	0.438276	0.755214	0.594113	0.044*	
C93	0.49743 (14)	0.73028 (9)	0.68752 (9)	0.0280 (4)	
O1	0.49191 (8)	0.24720 (6)	0.68828 (5)	0.0188 (2)	
O2	0.62194 (8)	0.17553 (6)	0.61614 (5)	0.0176 (2)	
O3	0.55712 (8)	0.11171 (6)	0.72348 (6)	0.0185 (2)	
O4	0.87391 (8)	0.05584 (6)	0.79991 (5)	0.0175 (2)	
O5	0.75557 (8)	0.08119 (6)	0.89670 (5)	0.0181 (2)	
O6	0.88858 (8)	0.15649 (6)	0.85846 (5)	0.0183 (2)	
O7	0.67415 (10)	−0.12389 (8)	0.93804 (8)	0.0405 (4)	
O8	0.83014 (9)	0.03127 (7)	0.53423 (6)	0.0279 (3)	
O9	0.87726 (9)	0.24214 (7)	0.63851 (6)	0.0277 (3)	
O10	0.68535 (9)	0.30387 (6)	0.87800 (5)	0.0192 (2)	
O11	0.78705 (8)	0.36703 (6)	0.77893 (6)	0.0203 (2)	
O12	0.60440 (8)	0.39513 (6)	0.78154 (5)	0.0187 (2)	
O13	0.66483 (9)	0.54450 (6)	0.71242 (6)	0.0248 (2)	
O14	0.51551 (10)	0.59357 (7)	0.78334 (7)	0.0284 (3)	
O15	0.57300 (10)	0.67130 (7)	0.67288 (6)	0.0292 (3)	
O16	0.36571 (10)	0.46844 (7)	0.55987 (7)	0.0304 (3)	
O17	0.90379 (9)	0.37875 (8)	0.53378 (7)	0.0309 (3)	
P1	0.59867 (3)	0.18802 (2)	0.69550 (2)	0.01480 (7)	
P2	0.80453 (3)	0.12767 (2)	0.82633 (2)	0.01506 (7)	
P3	0.69419 (3)	0.32232 (2)	0.79428 (2)	0.01578 (8)	
P4	0.54796 (4)	0.58775 (2)	0.70301 (2)	0.02573 (9)	
Rh1	0.70862 (2)	0.21807 (2)	0.75367 (2)	0.01398 (3)	
H1	0.6205 (17)	0.2069 (12)	0.8086 (12)	0.038 (6)*	
C94	0.81565 (17)	0.83512 (10)	0.63087 (10)	0.0601 (7)	
C95	0.79998 (15)	0.81955 (10)	0.56840 (11)	0.0711 (9)	
D95	0.736205	0.810153	0.561805	0.085*	
C96	0.8776 (2)	0.81775 (12)	0.51559 (9)	0.0833 (10)	
D96	0.866909	0.807113	0.472901	0.100*	
C97	0.97093 (17)	0.83150 (12)	0.52525 (15)	0.0923 (13)	
D97	1.023996	0.830271	0.489152	0.111*	
C98	0.98661 (15)	0.84707 (12)	0.58771 (18)	0.1020 (14)	
D98	1.050382	0.856467	0.594307	0.122*	
C99	0.9090 (2)	0.84887 (11)	0.64052 (13)	0.0929 (13)	
D99	0.919679	0.859507	0.683212	0.111*	
C100	0.7310 (3)	0.83474 (18)	0.6851 (2)	0.0997 (14)	
D10A	0.751266	0.846412	0.726276	0.150*	
D10B	0.671988	0.871746	0.668021	0.150*	
D10C	0.713345	0.785818	0.696971	0.150*	
C101	0.0868 (2)	0.72891 (19)	0.80410 (18)	0.0514 (11)	0.501 (3)
C102	0.08049 (19)	0.7024 (2)	0.74536 (17)	0.0455 (12)	0.501 (3)
D102	0.017986	0.713463	0.724560	0.055*	0.501 (3)
C103	0.1656 (2)	0.65960 (19)	0.71705 (14)	0.0407 (10)	0.501 (3)
D103	0.161232	0.641464	0.676906	0.049*	0.501 (3)
C104	0.2570 (2)	0.64337 (17)	0.74749 (16)	0.0389 (11)	0.501 (3)
D104	0.315178	0.614141	0.728140	0.047*	0.501 (3)
C105	0.2634 (2)	0.66991 (19)	0.80623 (17)	0.0454 (10)	0.501 (3)
D105	0.325880	0.658818	0.827029	0.054*	0.501 (3)
C106	0.1783 (3)	0.7127 (2)	0.83454 (15)	0.0488 (12)	0.501 (3)
D106	0.182634	0.730817	0.874685	0.059*	0.501 (3)
C107	−0.0019 (6)	0.7740 (5)	0.8369 (5)	0.090 (2)	0.501 (3)
D10D	0.018388	0.787119	0.877379	0.134*	0.501 (3)
D10E	−0.026704	0.819139	0.803505	0.134*	0.501 (3)
D10F	−0.055912	0.745668	0.851386	0.134*	0.501 (3)
C108	0.1602 (5)	0.6854 (4)	0.7735 (3)	0.0411 (15)	0.249 (3)
C109	0.1979 (4)	0.6863 (4)	0.8351 (4)	0.0405 (18)	0.249 (3)
D109	0.265558	0.662033	0.844174	0.049*	0.249 (3)
C110	0.1367 (6)	0.7228 (4)	0.8834 (3)	0.0535 (19)	0.249 (3)
D110	0.162495	0.723402	0.925498	0.064*	0.249 (3)
C111	0.0377 (6)	0.7583 (5)	0.8701 (4)	0.065 (2)	0.249 (3)
D111	−0.004105	0.783247	0.903113	0.078*	0.249 (3)
C112	0.0000 (5)	0.7574 (5)	0.8085 (4)	0.070 (2)	0.249 (3)
D112	−0.067642	0.781723	0.799404	0.084*	0.249 (3)
C113	0.0612 (5)	0.7210 (5)	0.7602 (3)	0.050 (2)	0.249 (3)
D113	0.035420	0.720354	0.718079	0.060*	0.249 (3)
C114	0.2247 (9)	0.6495 (7)	0.7252 (6)	0.049 (3)	0.249 (3)
D11A	0.188104	0.653322	0.684621	0.073*	0.249 (3)
D11B	0.283901	0.672750	0.710905	0.073*	0.249 (3)
D11C	0.247260	0.597402	0.746312	0.073*	0.249 (3)
C115	0.4748 (3)	0.02926 (18)	0.51899 (18)	0.0431 (10)	0.5
C116	0.5010 (3)	−0.04486 (19)	0.55014 (15)	0.0403 (17)	0.5
D116	0.493059	−0.059641	0.599103	0.048*	0.5
C117	0.5388 (3)	−0.09735 (16)	0.50964 (19)	0.0517 (11)	0.5
D117	0.556675	−0.148002	0.530924	0.062*	0.5
C118	0.5504 (4)	−0.07572 (19)	0.43799 (19)	0.064 (3)	0.5
D118	0.576206	−0.111593	0.410313	0.076*	0.5
C119	0.5242 (3)	−0.0016 (2)	0.40685 (15)	0.0586 (13)	0.5
D119	0.532120	0.013178	0.357879	0.070*	0.5
C120	0.4864 (3)	0.05089 (16)	0.44734 (19)	0.058 (2)	0.5
D120	0.468502	0.101540	0.426056	0.069*	0.5
C121	0.4341 (7)	0.0849 (5)	0.5626 (4)	0.061 (3)	0.5
D12A	0.419519	0.134131	0.532864	0.092*	0.5
D12B	0.371454	0.073755	0.589152	0.092*	0.5
D12C	0.484338	0.083650	0.594446	0.092*	0.5

### Atomic displacement parameters (Å^2^)

**Table:**

	*U*^11^	*U*^22^	*U*^33^	*U*^12^	*U*^13^	*U*^23^
C1	0.0165 (7)	0.0192 (7)	0.0213 (7)	0.0018 (6)	−0.0056 (6)	−0.0041 (6)
C2	0.0183 (8)	0.0314 (9)	0.0258 (8)	−0.0018 (7)	−0.0025 (6)	−0.0019 (7)
C3	0.0166 (8)	0.0467 (12)	0.0373 (10)	−0.0038 (8)	−0.0053 (7)	−0.0047 (9)
C4	0.0219 (9)	0.0551 (13)	0.0341 (10)	−0.0005 (8)	−0.0131 (8)	−0.0065 (9)
C5	0.0243 (9)	0.0393 (10)	0.0232 (8)	0.0014 (7)	−0.0073 (7)	−0.0034 (7)
C6	0.0190 (7)	0.0217 (8)	0.0216 (7)	0.0005 (6)	−0.0038 (6)	−0.0034 (6)
C7	0.0225 (8)	0.0207 (7)	0.0164 (7)	−0.0025 (6)	−0.0022 (6)	−0.0043 (6)
C8	0.0316 (9)	0.0218 (8)	0.0201 (8)	−0.0018 (7)	−0.0033 (7)	−0.0016 (6)
C9	0.0389 (10)	0.0259 (9)	0.0201 (8)	−0.0104 (7)	0.0016 (7)	−0.0018 (6)
C10	0.0281 (9)	0.0338 (9)	0.0216 (8)	−0.0106 (7)	0.0042 (7)	−0.0071 (7)
C11	0.0212 (8)	0.0271 (8)	0.0197 (7)	−0.0027 (6)	−0.0008 (6)	−0.0056 (6)
C12	0.0218 (7)	0.0185 (7)	0.0151 (7)	−0.0040 (6)	−0.0023 (6)	−0.0041 (6)
C13	0.0174 (7)	0.0166 (7)	0.0193 (7)	−0.0045 (6)	−0.0014 (6)	−0.0015 (6)
C14	0.0164 (7)	0.0220 (8)	0.0258 (8)	−0.0049 (6)	0.0009 (6)	−0.0011 (6)
C15	0.0223 (8)	0.0284 (9)	0.0303 (9)	−0.0091 (7)	0.0034 (7)	0.0051 (7)
C16	0.0242 (8)	0.0218 (8)	0.0299 (9)	−0.0057 (7)	−0.0042 (7)	0.0062 (7)
C17	0.0180 (7)	0.0184 (7)	0.0274 (8)	−0.0028 (6)	−0.0018 (6)	−0.0009 (6)
C18	0.0166 (7)	0.0171 (7)	0.0208 (7)	−0.0049 (6)	−0.0004 (6)	−0.0044 (6)
C19	0.0151 (7)	0.0142 (7)	0.0205 (7)	−0.0004 (5)	0.0006 (5)	−0.0039 (5)
C20	0.0172 (7)	0.0179 (7)	0.0234 (8)	−0.0019 (6)	−0.0025 (6)	−0.0056 (6)
C21	0.0218 (8)	0.0201 (7)	0.0202 (7)	−0.0002 (6)	−0.0011 (6)	−0.0064 (6)
C22	0.0177 (7)	0.0246 (8)	0.0202 (7)	−0.0006 (6)	0.0028 (6)	−0.0048 (6)
C23	0.0153 (7)	0.0178 (7)	0.0205 (7)	−0.0009 (6)	0.0000 (6)	−0.0021 (6)
C24	0.0167 (7)	0.0152 (7)	0.0173 (7)	−0.0005 (5)	−0.0007 (5)	−0.0024 (5)
C25	0.0149 (7)	0.0267 (9)	0.0319 (9)	−0.0047 (6)	0.0023 (6)	0.0017 (7)
C26	0.0192 (9)	0.0457 (12)	0.0532 (13)	−0.0067 (8)	0.0076 (8)	0.0114 (10)
C27	0.0225 (8)	0.0327 (9)	0.0301 (9)	0.0004 (7)	0.0014 (7)	−0.0061 (7)
C28	0.0192 (8)	0.0362 (10)	0.0440 (11)	−0.0088 (7)	−0.0043 (7)	−0.0082 (8)
C29	0.0314 (10)	0.0320 (10)	0.0506 (12)	−0.0069 (8)	−0.0152 (9)	0.0112 (9)
C30	0.0288 (9)	0.0401 (10)	0.0231 (8)	−0.0037 (8)	−0.0060 (7)	−0.0122 (7)
C31	0.0136 (7)	0.0274 (8)	0.0210 (7)	−0.0011 (6)	−0.0002 (6)	−0.0019 (6)
C32	0.0164 (8)	0.0303 (9)	0.0329 (9)	−0.0055 (7)	−0.0031 (6)	−0.0034 (7)
C33	0.0158 (8)	0.0458 (11)	0.0279 (9)	−0.0009 (7)	0.0032 (7)	−0.0066 (8)
C34	0.0168 (8)	0.0323 (9)	0.0277 (9)	0.0022 (7)	−0.0030 (6)	0.0014 (7)
C35	0.0202 (7)	0.0181 (7)	0.0148 (7)	−0.0028 (6)	0.0013 (6)	0.0014 (5)
C36	0.0199 (8)	0.0272 (8)	0.0202 (7)	−0.0056 (6)	0.0003 (6)	0.0033 (6)
C37	0.0217 (8)	0.0293 (9)	0.0263 (8)	0.0005 (7)	0.0065 (7)	0.0048 (7)
C38	0.0323 (9)	0.0279 (9)	0.0217 (8)	0.0004 (7)	0.0078 (7)	−0.0016 (7)
C39	0.0311 (9)	0.0269 (9)	0.0181 (7)	−0.0041 (7)	0.0013 (6)	−0.0035 (6)
C40	0.0219 (8)	0.0209 (7)	0.0151 (7)	−0.0036 (6)	0.0001 (6)	0.0010 (6)
C41	0.0211 (8)	0.0198 (7)	0.0182 (7)	−0.0031 (6)	−0.0035 (6)	−0.0022 (6)
C42	0.0294 (9)	0.0292 (9)	0.0167 (7)	−0.0065 (7)	−0.0042 (6)	−0.0012 (6)
C43	0.0300 (9)	0.0324 (9)	0.0231 (8)	−0.0048 (7)	−0.0120 (7)	−0.0006 (7)
C44	0.0206 (8)	0.0331 (9)	0.0311 (9)	−0.0009 (7)	−0.0091 (7)	−0.0060 (7)
C45	0.0196 (8)	0.0274 (8)	0.0232 (8)	−0.0035 (6)	−0.0021 (6)	−0.0060 (6)
C46	0.0212 (8)	0.0185 (7)	0.0169 (7)	−0.0043 (6)	−0.0054 (6)	−0.0016 (6)
C47	0.0188 (7)	0.0180 (7)	0.0187 (7)	−0.0004 (6)	−0.0059 (6)	−0.0006 (6)
C48	0.0314 (9)	0.0175 (7)	0.0149 (7)	−0.0044 (6)	−0.0025 (6)	−0.0028 (6)
C49	0.0288 (9)	0.0257 (8)	0.0209 (8)	−0.0039 (7)	−0.0003 (6)	−0.0023 (6)
C50	0.0421 (11)	0.0317 (10)	0.0258 (9)	−0.0027 (8)	0.0041 (8)	−0.0117 (7)
C51	0.0494 (12)	0.0390 (11)	0.0320 (10)	−0.0090 (9)	−0.0020 (9)	−0.0200 (8)
C52	0.0371 (10)	0.0382 (10)	0.0324 (9)	−0.0105 (8)	−0.0042 (8)	−0.0162 (8)
C53	0.0284 (9)	0.0228 (8)	0.0206 (7)	−0.0051 (7)	−0.0036 (6)	−0.0059 (6)
C54	0.0232 (8)	0.0223 (8)	0.0230 (8)	−0.0057 (6)	−0.0036 (6)	−0.0058 (6)
C55	0.0284 (9)	0.0358 (10)	0.0269 (9)	−0.0069 (8)	−0.0089 (7)	−0.0068 (7)
C56	0.0223 (9)	0.0382 (10)	0.0398 (10)	−0.0035 (8)	−0.0101 (8)	−0.0064 (8)
C57	0.0221 (8)	0.0372 (10)	0.0355 (10)	−0.0071 (7)	0.0018 (7)	−0.0106 (8)
C58	0.0230 (8)	0.0315 (9)	0.0239 (8)	−0.0089 (7)	−0.0006 (6)	−0.0061 (7)
C59	0.0202 (8)	0.0198 (7)	0.0232 (8)	−0.0065 (6)	−0.0044 (6)	−0.0025 (6)
C60	0.0193 (7)	0.0151 (7)	0.0187 (7)	0.0000 (6)	−0.0028 (6)	−0.0029 (5)
C61	0.0195 (8)	0.0187 (7)	0.0242 (8)	−0.0012 (6)	0.0011 (6)	−0.0033 (6)
C62	0.0168 (7)	0.0230 (8)	0.0314 (9)	−0.0011 (6)	−0.0029 (6)	−0.0025 (7)
C63	0.0213 (8)	0.0196 (8)	0.0264 (8)	0.0003 (6)	−0.0070 (6)	−0.0021 (6)
C64	0.0233 (8)	0.0174 (7)	0.0198 (7)	−0.0018 (6)	−0.0022 (6)	−0.0022 (6)
C65	0.0181 (7)	0.0138 (7)	0.0203 (7)	0.0008 (5)	−0.0010 (6)	−0.0048 (5)
C66	0.0196 (7)	0.0154 (7)	0.0161 (7)	−0.0008 (6)	−0.0013 (6)	0.0005 (5)
C67	0.0216 (8)	0.0204 (7)	0.0171 (7)	−0.0007 (6)	−0.0023 (6)	−0.0026 (6)
C68	0.0225 (8)	0.0229 (8)	0.0182 (7)	0.0022 (6)	0.0011 (6)	−0.0010 (6)
C69	0.0188 (8)	0.0255 (8)	0.0270 (8)	−0.0043 (6)	0.0008 (6)	0.0008 (7)
C70	0.0245 (8)	0.0200 (8)	0.0261 (8)	−0.0055 (6)	−0.0029 (6)	−0.0004 (6)
C71	0.0227 (8)	0.0167 (7)	0.0201 (7)	−0.0011 (6)	−0.0001 (6)	−0.0024 (6)
C72	0.0209 (8)	0.0284 (9)	0.0260 (8)	−0.0022 (7)	0.0053 (6)	−0.0027 (7)
C73	0.0344 (10)	0.0263 (9)	0.0299 (9)	−0.0084 (7)	0.0083 (7)	−0.0023 (7)
C74	0.0230 (10)	0.0718 (16)	0.0390 (11)	−0.0099 (10)	0.0076 (8)	0.0013 (11)
C75	0.0404 (11)	0.0264 (9)	0.0278 (9)	−0.0005 (8)	0.0104 (8)	−0.0068 (7)
C76	0.0265 (10)	0.0479 (12)	0.0464 (12)	−0.0111 (9)	−0.0171 (9)	0.0094 (10)
C77	0.0230 (9)	0.0522 (13)	0.0422 (11)	0.0027 (9)	0.0082 (8)	−0.0141 (10)
C78	0.0283 (9)	0.0268 (9)	0.0442 (11)	−0.0103 (7)	−0.0043 (8)	−0.0092 (8)
C79	0.0417 (12)	0.0263 (10)	0.0726 (16)	−0.0134 (9)	−0.0074 (11)	−0.0084 (10)
C80	0.0299 (10)	0.0455 (12)	0.0644 (15)	−0.0159 (9)	−0.0051 (10)	−0.0167 (11)
C81	0.0436 (12)	0.0477 (12)	0.0425 (11)	−0.0161 (10)	−0.0087 (9)	−0.0179 (10)
C82	0.0288 (9)	0.0263 (9)	0.0257 (8)	−0.0045 (7)	0.0071 (7)	−0.0066 (7)
C83	0.0366 (10)	0.0301 (10)	0.0313 (9)	−0.0028 (8)	0.0017 (8)	−0.0003 (8)
C84	0.0426 (12)	0.0470 (12)	0.0328 (10)	−0.0039 (10)	−0.0045 (9)	−0.0040 (9)
C85	0.0455 (12)	0.0494 (13)	0.0383 (11)	−0.0102 (10)	−0.0028 (9)	−0.0165 (10)
C86	0.0427 (11)	0.0308 (10)	0.0362 (10)	−0.0071 (8)	0.0045 (8)	−0.0128 (8)
C87	0.0321 (9)	0.0260 (9)	0.0256 (8)	−0.0025 (7)	0.0063 (7)	−0.0075 (7)
C88	0.0353 (10)	0.0200 (8)	0.0293 (9)	−0.0021 (7)	0.0047 (7)	−0.0042 (7)
C89	0.0521 (13)	0.0257 (9)	0.0353 (10)	0.0039 (9)	0.0041 (9)	−0.0087 (8)
C90	0.0550 (14)	0.0264 (10)	0.0487 (13)	0.0119 (9)	0.0026 (11)	−0.0067 (9)
C91	0.0495 (13)	0.0300 (10)	0.0464 (12)	0.0066 (9)	−0.0095 (10)	0.0001 (9)
C92	0.0474 (12)	0.0261 (9)	0.0331 (10)	−0.0021 (8)	−0.0035 (9)	−0.0042 (8)
C93	0.0337 (9)	0.0170 (8)	0.0297 (9)	−0.0019 (7)	0.0045 (7)	−0.0046 (7)
O1	0.0152 (5)	0.0217 (5)	0.0180 (5)	0.0017 (4)	−0.0036 (4)	−0.0052 (4)
O2	0.0187 (5)	0.0173 (5)	0.0153 (5)	−0.0012 (4)	−0.0012 (4)	−0.0029 (4)
O3	0.0155 (5)	0.0184 (5)	0.0206 (5)	−0.0047 (4)	−0.0030 (4)	0.0001 (4)
O4	0.0153 (5)	0.0191 (5)	0.0163 (5)	0.0000 (4)	−0.0016 (4)	−0.0030 (4)
O5	0.0183 (5)	0.0192 (5)	0.0157 (5)	−0.0046 (4)	0.0003 (4)	−0.0014 (4)
O6	0.0188 (5)	0.0211 (5)	0.0146 (5)	−0.0059 (4)	−0.0036 (4)	0.0002 (4)
O7	0.0272 (7)	0.0363 (8)	0.0433 (8)	−0.0047 (6)	−0.0028 (6)	0.0207 (6)
O8	0.0237 (6)	0.0411 (7)	0.0192 (6)	−0.0031 (5)	−0.0011 (5)	−0.0104 (5)
O9	0.0204 (6)	0.0351 (7)	0.0226 (6)	−0.0034 (5)	0.0033 (5)	−0.0002 (5)
O10	0.0252 (6)	0.0191 (5)	0.0142 (5)	−0.0065 (4)	−0.0021 (4)	−0.0027 (4)
O11	0.0199 (5)	0.0211 (5)	0.0198 (5)	−0.0066 (4)	−0.0037 (4)	0.0001 (4)
O12	0.0197 (5)	0.0181 (5)	0.0167 (5)	−0.0002 (4)	−0.0019 (4)	−0.0038 (4)
O13	0.0244 (6)	0.0207 (6)	0.0305 (6)	−0.0018 (5)	−0.0011 (5)	−0.0111 (5)
O14	0.0298 (7)	0.0222 (6)	0.0313 (6)	−0.0051 (5)	0.0049 (5)	−0.0058 (5)
O15	0.0359 (7)	0.0197 (6)	0.0277 (6)	−0.0005 (5)	0.0055 (5)	−0.0056 (5)
O16	0.0240 (6)	0.0353 (7)	0.0297 (6)	−0.0049 (5)	−0.0113 (5)	0.0021 (5)
O17	0.0225 (6)	0.0384 (7)	0.0287 (6)	0.0015 (5)	0.0054 (5)	−0.0121 (5)
P1	0.01251 (17)	0.01612 (18)	0.01463 (17)	−0.00149 (13)	−0.00114 (13)	−0.00211 (13)
P2	0.01368 (17)	0.01664 (18)	0.01351 (16)	−0.00189 (14)	−0.00121 (13)	−0.00131 (13)
P3	0.01680 (18)	0.01565 (18)	0.01418 (17)	−0.00267 (14)	−0.00147 (13)	−0.00195 (13)
P4	0.0258 (2)	0.0190 (2)	0.0313 (2)	0.00000 (17)	−0.00118 (18)	−0.00767 (17)
Rh1	0.01282 (6)	0.01477 (6)	0.01325 (6)	−0.00160 (4)	−0.00098 (4)	−0.00170 (4)
C94	0.0768 (19)	0.0263 (11)	0.0703 (18)	−0.0067 (12)	0.0020 (15)	−0.0032 (11)
C95	0.106 (3)	0.0379 (14)	0.0603 (17)	0.0017 (15)	−0.0111 (17)	−0.0056 (12)
C96	0.104 (3)	0.0506 (17)	0.079 (2)	−0.0046 (18)	0.007 (2)	0.0022 (15)
C97	0.082 (3)	0.0433 (17)	0.133 (4)	−0.0092 (16)	0.021 (2)	0.0008 (19)
C98	0.086 (3)	0.051 (2)	0.168 (5)	−0.0130 (18)	−0.019 (3)	−0.017 (2)
C99	0.094 (3)	0.0404 (16)	0.148 (4)	−0.0042 (17)	−0.042 (3)	−0.016 (2)
C100	0.134 (3)	0.0449 (17)	0.099 (3)	−0.0099 (19)	0.050 (2)	−0.0101 (17)
C101	0.042 (2)	0.051 (2)	0.065 (2)	−0.0079 (18)	−0.001 (2)	−0.021 (2)
C102	0.030 (2)	0.055 (3)	0.053 (3)	−0.0035 (19)	−0.0101 (19)	−0.014 (2)
C103	0.036 (2)	0.055 (2)	0.034 (2)	−0.015 (2)	−0.0036 (18)	−0.0082 (18)
C104	0.019 (2)	0.048 (2)	0.040 (2)	−0.0048 (18)	−0.0023 (17)	0.010 (2)
C105	0.033 (2)	0.052 (2)	0.050 (2)	−0.0132 (19)	−0.0098 (18)	−0.0017 (19)
C106	0.051 (3)	0.052 (3)	0.054 (2)	−0.019 (2)	−0.007 (2)	−0.022 (2)
C107	0.065 (4)	0.096 (5)	0.126 (6)	0.002 (4)	−0.004 (4)	−0.078 (5)
C108	0.035 (3)	0.044 (3)	0.044 (3)	−0.009 (3)	−0.009 (3)	−0.003 (3)
C109	0.043 (4)	0.037 (4)	0.046 (3)	−0.018 (3)	−0.015 (3)	−0.001 (3)
C110	0.067 (4)	0.051 (4)	0.054 (4)	−0.023 (3)	−0.012 (4)	−0.019 (3)
C111	0.078 (5)	0.070 (5)	0.054 (4)	0.003 (4)	−0.020 (4)	−0.037 (4)
C112	0.065 (4)	0.080 (5)	0.059 (4)	0.011 (4)	−0.018 (4)	−0.022 (4)
C113	0.047 (4)	0.061 (4)	0.040 (4)	0.005 (3)	−0.013 (3)	−0.019 (3)
C114	0.029 (5)	0.059 (5)	0.040 (5)	0.007 (5)	0.002 (4)	0.009 (4)
C115	0.032 (2)	0.054 (3)	0.055 (2)	−0.0189 (19)	0.0003 (18)	−0.027 (2)
C116	0.040 (3)	0.044 (3)	0.043 (3)	−0.014 (3)	−0.011 (3)	−0.011 (3)
C117	0.051 (3)	0.042 (2)	0.065 (3)	−0.013 (2)	−0.002 (2)	−0.012 (2)
C118	0.061 (4)	0.064 (5)	0.070 (5)	−0.021 (4)	0.015 (4)	−0.027 (4)
C119	0.069 (3)	0.059 (3)	0.050 (3)	−0.021 (3)	0.015 (2)	−0.018 (2)
C120	0.053 (4)	0.058 (4)	0.060 (5)	−0.019 (3)	0.017 (3)	−0.013 (4)
C121	0.064 (4)	0.065 (5)	0.070 (5)	−0.016 (4)	−0.001 (4)	−0.045 (4)

### Geometric parameters (Å, º)

**Table:**

C1—C2	1.384 (2)	C67—C68	1.380 (2)
C1—O1	1.3925 (18)	C67—H67	0.9500
C1—C6	1.397 (2)	C68—O17	1.375 (2)
C2—C3	1.388 (2)	C68—C69	1.384 (3)
C2—H2	0.9500	C69—C70	1.396 (2)
C3—C4	1.381 (3)	C69—H69	0.9500
C3—H3	0.9500	C70—C71	1.400 (2)
C4—C5	1.379 (3)	C70—C78	1.546 (2)
C4—H4	0.9500	C71—O13	1.3972 (19)
C5—C6	1.400 (2)	C72—C75	1.529 (3)
C5—H5	0.9500	C72—C74	1.534 (3)
C6—C7	1.475 (2)	C72—C73	1.535 (3)
C7—C12	1.393 (2)	C73—H73A	0.9800
C7—C8	1.401 (2)	C73—H73B	0.9800
C8—C9	1.381 (3)	C73—H73C	0.9800
C8—H8	0.9500	C74—H74A	0.9800
C9—C10	1.387 (3)	C74—H74B	0.9800
C9—H9	0.9500	C74—H74C	0.9800
C10—C11	1.387 (2)	C75—H75A	0.9800
C10—H10	0.9500	C75—H75B	0.9800
C11—C12	1.382 (2)	C75—H75C	0.9800
C11—H11	0.9500	C76—O16	1.423 (2)
C12—O2	1.3911 (18)	C76—H76A	0.9800
C13—C18	1.392 (2)	C76—H76B	0.9800
C13—O3	1.3974 (18)	C76—H76C	0.9800
C13—C14	1.416 (2)	C77—O17	1.411 (2)
C14—C15	1.386 (2)	C77—H77A	0.9800
C14—C25	1.538 (2)	C77—H77B	0.9800
C15—C16	1.387 (2)	C77—H77C	0.9800
C15—H15	0.9500	C78—C79	1.532 (3)
C16—C17	1.375 (2)	C78—C81	1.535 (3)
C16—O7	1.376 (2)	C78—C80	1.537 (3)
C17—C18	1.403 (2)	C79—H79A	0.9800
C17—H17	0.9500	C79—H79B	0.9800
C18—C19	1.493 (2)	C79—H79C	0.9800
C19—C20	1.395 (2)	C80—H80A	0.9800
C19—C24	1.397 (2)	C80—H80B	0.9800
C20—C21	1.381 (2)	C80—H80C	0.9800
C20—H20	0.9500	C81—H81A	0.9800
C21—O8	1.3682 (19)	C81—H81B	0.9800
C21—C22	1.392 (2)	C81—H81C	0.9800
C22—C23	1.391 (2)	C82—C83	1.382 (3)
C22—H22	0.9500	C82—C87	1.395 (3)
C23—C24	1.404 (2)	C82—O14	1.396 (2)
C23—C31	1.540 (2)	C83—C84	1.379 (3)
C24—O4	1.4040 (18)	C83—H83	0.9500
C25—C27	1.534 (3)	C84—C85	1.382 (3)
C25—C28	1.534 (3)	C84—H84	0.9500
C25—C26	1.538 (2)	C85—C86	1.380 (3)
C26—H26A	0.9800	C85—H85	0.9500
C26—H26B	0.9800	C86—C87	1.394 (3)
C26—H26C	0.9800	C86—H86	0.9500
C27—H27A	0.9800	C87—C88	1.479 (3)
C27—H27B	0.9800	C88—C93	1.392 (3)
C27—H27C	0.9800	C88—C89	1.396 (3)
C28—H28A	0.9800	C89—C90	1.382 (3)
C28—H28B	0.9800	C89—H89	0.9500
C28—H28C	0.9800	C90—C91	1.383 (3)
C29—O7	1.406 (2)	C90—H90	0.9500
C29—H29A	0.9800	C91—C92	1.388 (3)
C29—H29B	0.9800	C91—H91	0.9500
C29—H29C	0.9800	C92—C93	1.378 (3)
C30—O8	1.424 (2)	C92—H92	0.9500
C30—H30A	0.9800	C93—O15	1.396 (2)
C30—H30B	0.9800	O1—P1	1.6237 (11)
C30—H30C	0.9800	O2—P1	1.6289 (11)
C31—C32	1.530 (2)	O3—P1	1.6258 (11)
C31—C33	1.532 (2)	O4—P2	1.6271 (11)
C31—C34	1.535 (2)	O5—P2	1.6270 (11)
C32—H32A	0.9800	O6—P2	1.6179 (11)
C32—H32B	0.9800	O10—P3	1.6168 (11)
C32—H32C	0.9800	O11—P3	1.6293 (11)
C33—H33A	0.9800	O12—P3	1.6221 (11)
C33—H33B	0.9800	O13—P4	1.6269 (13)
C33—H33C	0.9800	O14—P4	1.6237 (13)
C34—H34A	0.9800	O15—P4	1.6579 (13)
C34—H34B	0.9800	P1—Rh1	2.2325 (4)
C34—H34C	0.9800	P2—Rh1	2.2572 (4)
C35—C36	1.384 (2)	P3—Rh1	2.2600 (4)
C35—O5	1.3922 (19)	Rh1—H1	1.53 (2)
C35—C40	1.392 (2)	C94—C95	1.3900
C36—C37	1.387 (2)	C94—C99	1.3900
C36—H36	0.9500	C94—C100	1.472 (4)
C37—C38	1.383 (3)	C95—C96	1.3900
C37—H37	0.9500	C95—D95	0.9500
C38—C39	1.382 (3)	C96—C97	1.3900
C38—H38	0.9500	C96—D96	0.9500
C39—C40	1.397 (2)	C97—C98	1.3900
C39—H39	0.9500	C97—D97	0.9500
C40—C41	1.475 (2)	C98—C99	1.3900
C41—C46	1.393 (2)	C98—D98	0.9500
C41—C42	1.398 (2)	C99—D99	0.9500
C42—C43	1.379 (3)	C100—D10A	0.9800
C42—H42	0.9500	C100—D10B	0.9800
C43—C44	1.384 (3)	C100—D10C	0.9800
C43—H43	0.9500	C101—C102	1.3900
C44—C45	1.387 (2)	C101—C106	1.3900
C44—H44	0.9500	C101—C107	1.483 (7)
C45—C46	1.383 (2)	C102—C103	1.3900
C45—H45	0.9500	C102—D102	0.9500
C46—O6	1.3935 (18)	C103—C104	1.3900
C47—O9	1.135 (2)	C103—D103	0.9500
C47—Rh1	1.9227 (16)	C104—C105	1.3900
C48—C49	1.383 (2)	C104—D104	0.9500
C48—C53	1.392 (2)	C105—C106	1.3900
C48—O10	1.3943 (18)	C105—D105	0.9500
C49—C50	1.388 (3)	C106—D106	0.9500
C49—H49	0.9500	C107—D10D	0.9800
C50—C51	1.381 (3)	C107—D10E	0.9800
C50—H50	0.9500	C107—D10F	0.9800
C51—C52	1.379 (3)	C108—C109	1.3900
C51—H51	0.9500	C108—C113	1.3900
C52—C53	1.403 (2)	C108—C114	1.403 (12)
C52—H52	0.9500	C109—C110	1.3900
C53—C54	1.476 (2)	C109—D109	0.9500
C54—C55	1.397 (2)	C110—C111	1.3900
C54—C59	1.398 (2)	C110—D110	0.9500
C55—C56	1.379 (3)	C111—C112	1.3900
C55—H55	0.9500	C111—D111	0.9500
C56—C57	1.383 (3)	C112—C113	1.3900
C56—H56	0.9500	C112—D112	0.9500
C57—C58	1.388 (3)	C113—D113	0.9500
C57—H57	0.9500	C114—D11A	0.9800
C58—C59	1.380 (2)	C114—D11B	0.9800
C58—H58	0.9500	C114—D11C	0.9800
C59—O11	1.3892 (19)	C115—C116	1.3900
C60—C61	1.400 (2)	C115—C120	1.3900
C60—C65	1.400 (2)	C115—C121	1.484 (7)
C60—O12	1.4026 (18)	C116—C117	1.3900
C61—C62	1.399 (2)	C116—D116	0.9500
C61—C72	1.541 (2)	C117—C118	1.3900
C62—C63	1.383 (2)	C117—D117	0.9500
C62—H62	0.9500	C118—C119	1.3900
C63—O16	1.375 (2)	C118—D118	0.9500
C63—C64	1.383 (2)	C119—C120	1.3900
C64—C65	1.388 (2)	C119—D119	0.9500
C64—H64	0.9500	C120—D120	0.9500
C65—C66	1.490 (2)	C121—D12A	0.9800
C66—C67	1.387 (2)	C121—D12B	0.9800
C66—C71	1.401 (2)	C121—D12C	0.9800
			
C2—C1—O1	117.29 (14)	C70—C71—C66	121.40 (15)
C2—C1—C6	121.52 (15)	C75—C72—C74	107.51 (17)
O1—C1—C6	120.88 (14)	C75—C72—C73	110.06 (16)
C1—C2—C3	119.71 (16)	C74—C72—C73	106.79 (16)
C1—C2—H2	120.1	C75—C72—C61	109.59 (14)
C3—C2—H2	120.1	C74—C72—C61	111.42 (15)
C4—C3—C2	119.86 (17)	C73—C72—C61	111.36 (14)
C4—C3—H3	120.1	C72—C73—H73A	109.5
C2—C3—H3	120.1	C72—C73—H73B	109.5
C5—C4—C3	120.09 (17)	H73A—C73—H73B	109.5
C5—C4—H4	120.0	C72—C73—H73C	109.5
C3—C4—H4	120.0	H73A—C73—H73C	109.5
C4—C5—C6	121.49 (17)	H73B—C73—H73C	109.5
C4—C5—H5	119.3	C72—C74—H74A	109.5
C6—C5—H5	119.3	C72—C74—H74B	109.5
C1—C6—C5	117.31 (15)	H74A—C74—H74B	109.5
C1—C6—C7	123.26 (14)	C72—C74—H74C	109.5
C5—C6—C7	119.39 (15)	H74A—C74—H74C	109.5
C12—C7—C8	117.42 (15)	H74B—C74—H74C	109.5
C12—C7—C6	121.04 (14)	C72—C75—H75A	109.5
C8—C7—C6	121.22 (15)	C72—C75—H75B	109.5
C9—C8—C7	121.06 (16)	H75A—C75—H75B	109.5
C9—C8—H8	119.5	C72—C75—H75C	109.5
C7—C8—H8	119.5	H75A—C75—H75C	109.5
C8—C9—C10	120.17 (16)	H75B—C75—H75C	109.5
C8—C9—H9	119.9	O16—C76—H76A	109.5
C10—C9—H9	119.9	O16—C76—H76B	109.5
C11—C10—C9	119.85 (16)	H76A—C76—H76B	109.5
C11—C10—H10	120.1	O16—C76—H76C	109.5
C9—C10—H10	120.1	H76A—C76—H76C	109.5
C12—C11—C10	119.42 (16)	H76B—C76—H76C	109.5
C12—C11—H11	120.3	O17—C77—H77A	109.5
C10—C11—H11	120.3	O17—C77—H77B	109.5
C11—C12—O2	118.51 (14)	H77A—C77—H77B	109.5
C11—C12—C7	121.99 (15)	O17—C77—H77C	109.5
O2—C12—C7	119.28 (14)	H77A—C77—H77C	109.5
C18—C13—O3	120.63 (13)	H77B—C77—H77C	109.5
C18—C13—C14	121.01 (14)	C79—C78—C81	110.80 (18)
O3—C13—C14	118.13 (13)	C79—C78—C80	107.26 (17)
C15—C14—C13	117.25 (15)	C81—C78—C80	106.73 (18)
C15—C14—C25	119.27 (14)	C79—C78—C70	109.91 (16)
C13—C14—C25	123.43 (14)	C81—C78—C70	110.40 (15)
C14—C15—C16	122.21 (16)	C80—C78—C70	111.65 (16)
C14—C15—H15	118.9	C78—C79—H79A	109.5
C16—C15—H15	118.9	C78—C79—H79B	109.5
C17—C16—O7	125.11 (16)	H79A—C79—H79B	109.5
C17—C16—C15	119.86 (15)	C78—C79—H79C	109.5
O7—C16—C15	115.02 (15)	H79A—C79—H79C	109.5
C16—C17—C18	120.09 (15)	H79B—C79—H79C	109.5
C16—C17—H17	120.0	C78—C80—H80A	109.5
C18—C17—H17	120.0	C78—C80—H80B	109.5
C13—C18—C17	119.42 (14)	H80A—C80—H80B	109.5
C13—C18—C19	124.85 (14)	C78—C80—H80C	109.5
C17—C18—C19	115.54 (14)	H80A—C80—H80C	109.5
C20—C19—C24	119.47 (14)	H80B—C80—H80C	109.5
C20—C19—C18	119.25 (14)	C78—C81—H81A	109.5
C24—C19—C18	120.82 (14)	C78—C81—H81B	109.5
C21—C20—C19	119.29 (14)	H81A—C81—H81B	109.5
C21—C20—H20	120.4	C78—C81—H81C	109.5
C19—C20—H20	120.4	H81A—C81—H81C	109.5
O8—C21—C20	124.31 (15)	H81B—C81—H81C	109.5
O8—C21—C22	115.53 (14)	C83—C82—C87	122.22 (18)
C20—C21—C22	120.12 (15)	C83—C82—O14	118.04 (16)
C23—C22—C21	122.30 (15)	C87—C82—O14	119.58 (17)
C23—C22—H22	118.8	C84—C83—C82	119.14 (19)
C21—C22—H22	118.8	C84—C83—H83	120.4
C22—C23—C24	116.31 (14)	C82—C83—H83	120.4
C22—C23—C31	120.93 (14)	C83—C84—C85	119.9 (2)
C24—C23—C31	122.74 (14)	C83—C84—H84	120.1
C19—C24—O4	118.45 (13)	C85—C84—H84	120.1
C19—C24—C23	121.96 (14)	C86—C85—C84	120.7 (2)
O4—C24—C23	119.58 (13)	C86—C85—H85	119.7
C27—C25—C28	111.02 (15)	C84—C85—H85	119.7
C27—C25—C14	110.16 (14)	C85—C86—C87	120.69 (19)
C28—C25—C14	109.83 (15)	C85—C86—H86	119.7
C27—C25—C26	107.27 (16)	C87—C86—H86	119.7
C28—C25—C26	106.76 (16)	C86—C87—C82	117.38 (18)
C14—C25—C26	111.75 (14)	C86—C87—C88	122.22 (17)
C25—C26—H26A	109.5	C82—C87—C88	120.31 (17)
C25—C26—H26B	109.5	C93—C88—C89	117.54 (18)
H26A—C26—H26B	109.5	C93—C88—C87	121.07 (16)
C25—C26—H26C	109.5	C89—C88—C87	121.34 (17)
H26A—C26—H26C	109.5	C90—C89—C88	120.9 (2)
H26B—C26—H26C	109.5	C90—C89—H89	119.5
C25—C27—H27A	109.5	C88—C89—H89	119.5
C25—C27—H27B	109.5	C89—C90—C91	120.22 (19)
H27A—C27—H27B	109.5	C89—C90—H90	119.9
C25—C27—H27C	109.5	C91—C90—H90	119.9
H27A—C27—H27C	109.5	C90—C91—C92	120.0 (2)
H27B—C27—H27C	109.5	C90—C91—H91	120.0
C25—C28—H28A	109.5	C92—C91—H91	120.0
C25—C28—H28B	109.5	C93—C92—C91	119.14 (19)
H28A—C28—H28B	109.5	C93—C92—H92	120.4
C25—C28—H28C	109.5	C91—C92—H92	120.4
H28A—C28—H28C	109.5	C92—C93—C88	122.14 (17)
H28B—C28—H28C	109.5	C92—C93—O15	119.42 (17)
O7—C29—H29A	109.5	C88—C93—O15	118.40 (16)
O7—C29—H29B	109.5	C1—O1—P1	126.60 (10)
H29A—C29—H29B	109.5	C12—O2—P1	119.87 (9)
O7—C29—H29C	109.5	C13—O3—P1	128.64 (10)
H29A—C29—H29C	109.5	C24—O4—P2	120.30 (9)
H29B—C29—H29C	109.5	C35—O5—P2	115.59 (9)
O8—C30—H30A	109.5	C46—O6—P2	126.53 (10)
O8—C30—H30B	109.5	C16—O7—C29	117.25 (15)
H30A—C30—H30B	109.5	C21—O8—C30	116.22 (13)
O8—C30—H30C	109.5	C48—O10—P3	125.20 (10)
H30A—C30—H30C	109.5	C59—O11—P3	120.47 (10)
H30B—C30—H30C	109.5	C60—O12—P3	125.01 (10)
C32—C31—C33	107.86 (14)	C71—O13—P4	127.26 (11)
C32—C31—C34	110.15 (14)	C82—O14—P4	123.17 (11)
C33—C31—C34	106.63 (14)	C93—O15—P4	116.16 (11)
C32—C31—C23	110.42 (13)	C63—O16—C76	117.26 (15)
C33—C31—C23	111.20 (14)	C68—O17—C77	117.24 (15)
C34—C31—C23	110.47 (13)	O1—P1—O3	100.33 (6)
C31—C32—H32A	109.5	O1—P1—O2	99.78 (6)
C31—C32—H32B	109.5	O3—P1—O2	93.64 (6)
H32A—C32—H32B	109.5	O1—P1—Rh1	113.71 (4)
C31—C32—H32C	109.5	O3—P1—Rh1	120.63 (4)
H32A—C32—H32C	109.5	O2—P1—Rh1	124.12 (4)
H32B—C32—H32C	109.5	O6—P2—O5	99.93 (6)
C31—C33—H33A	109.5	O6—P2—O4	100.91 (6)
C31—C33—H33B	109.5	O5—P2—O4	95.49 (6)
H33A—C33—H33B	109.5	O6—P2—Rh1	113.09 (4)
C31—C33—H33C	109.5	O5—P2—Rh1	122.18 (4)
H33A—C33—H33C	109.5	O4—P2—Rh1	121.13 (4)
H33B—C33—H33C	109.5	O10—P3—O12	99.18 (6)
C31—C34—H34A	109.5	O10—P3—O11	100.23 (6)
C31—C34—H34B	109.5	O12—P3—O11	95.66 (6)
H34A—C34—H34B	109.5	O10—P3—Rh1	110.13 (4)
C31—C34—H34C	109.5	O12—P3—Rh1	126.79 (4)
H34A—C34—H34C	109.5	O11—P3—Rh1	120.20 (5)
H34B—C34—H34C	109.5	O14—P4—O13	97.63 (7)
C36—C35—O5	118.61 (14)	O14—P4—O15	99.05 (6)
C36—C35—C40	122.36 (15)	O13—P4—O15	96.77 (7)
O5—C35—C40	118.99 (14)	C47—Rh1—P1	99.54 (5)
C35—C36—C37	118.95 (16)	C47—Rh1—P2	97.23 (5)
C35—C36—H36	120.5	P1—Rh1—P2	118.789 (15)
C37—C36—H36	120.5	C47—Rh1—P3	98.70 (5)
C38—C37—C36	119.99 (16)	P1—Rh1—P3	128.401 (15)
C38—C37—H37	120.0	P2—Rh1—P3	106.081 (15)
C36—C37—H37	120.0	C47—Rh1—H1	176.5 (8)
C39—C38—C37	120.34 (16)	P1—Rh1—H1	77.5 (8)
C39—C38—H38	119.8	P2—Rh1—H1	85.8 (8)
C37—C38—H38	119.8	P3—Rh1—H1	82.0 (8)
C38—C39—C40	121.05 (17)	C95—C94—C99	120.0
C38—C39—H39	119.5	C95—C94—C100	117.2 (3)
C40—C39—H39	119.5	C99—C94—C100	122.8 (3)
C35—C40—C39	117.27 (15)	C94—C95—C96	120.0
C35—C40—C41	120.93 (14)	C94—C95—D95	120.0
C39—C40—C41	121.76 (15)	C96—C95—D95	120.0
C46—C41—C42	117.55 (15)	C97—C96—C95	120.0
C46—C41—C40	122.54 (14)	C97—C96—D96	120.0
C42—C41—C40	119.87 (14)	C95—C96—D96	120.0
C43—C42—C41	121.49 (16)	C98—C97—C96	120.0
C43—C42—H42	119.3	C98—C97—D97	120.0
C41—C42—H42	119.3	C96—C97—D97	120.0
C42—C43—C44	119.69 (16)	C97—C98—C99	120.0
C42—C43—H43	120.2	C97—C98—D98	120.0
C44—C43—H43	120.2	C99—C98—D98	120.0
C43—C44—C45	120.24 (16)	C98—C99—C94	120.0
C43—C44—H44	119.9	C98—C99—D99	120.0
C45—C44—H44	119.9	C94—C99—D99	120.0
C46—C45—C44	119.44 (16)	C94—C100—D10A	109.5
C46—C45—H45	120.3	C94—C100—D10B	109.5
C44—C45—H45	120.3	D10A—C100—D10B	109.5
C45—C46—C41	121.59 (14)	C94—C100—D10C	109.5
C45—C46—O6	117.75 (14)	D10A—C100—D10C	109.5
C41—C46—O6	120.42 (14)	D10B—C100—D10C	109.5
O9—C47—Rh1	178.18 (14)	C102—C101—C106	120.0
C49—C48—C53	122.03 (15)	C102—C101—C107	122.2 (4)
C49—C48—O10	117.51 (15)	C106—C101—C107	117.8 (4)
C53—C48—O10	120.07 (14)	C103—C102—C101	120.0
C48—C49—C50	119.49 (17)	C103—C102—D102	120.0
C48—C49—H49	120.3	C101—C102—D102	120.0
C50—C49—H49	120.3	C102—C103—C104	120.0
C51—C50—C49	119.80 (18)	C102—C103—D103	120.0
C51—C50—H50	120.1	C104—C103—D103	120.0
C49—C50—H50	120.1	C105—C104—C103	120.0
C52—C51—C50	120.24 (18)	C105—C104—D104	120.0
C52—C51—H51	119.9	C103—C104—D104	120.0
C50—C51—H51	119.9	C104—C105—C106	120.0
C51—C52—C53	121.33 (18)	C104—C105—D105	120.0
C51—C52—H52	119.3	C106—C105—D105	120.0
C53—C52—H52	119.3	C105—C106—C101	120.0
C48—C53—C52	117.10 (16)	C105—C106—D106	120.0
C48—C53—C54	122.85 (15)	C101—C106—D106	120.0
C52—C53—C54	120.03 (16)	C101—C107—D10D	109.5
C55—C54—C59	117.24 (16)	C101—C107—D10E	109.5
C55—C54—C53	121.13 (15)	D10D—C107—D10E	109.5
C59—C54—C53	121.56 (15)	C101—C107—D10F	109.5
C56—C55—C54	121.30 (17)	D10D—C107—D10F	109.5
C56—C55—H55	119.4	D10E—C107—D10F	109.5
C54—C55—H55	119.4	C109—C108—C113	120.0
C55—C56—C57	120.16 (17)	C109—C108—C114	118.5 (7)
C55—C56—H56	119.9	C113—C108—C114	121.5 (7)
C57—C56—H56	119.9	C108—C109—C110	120.0
C56—C57—C58	119.96 (17)	C108—C109—D109	120.0
C56—C57—H57	120.0	C110—C109—D109	120.0
C58—C57—H57	120.0	C109—C110—C111	120.0
C59—C58—C57	119.29 (16)	C109—C110—D110	120.0
C59—C58—H58	120.4	C111—C110—D110	120.0
C57—C58—H58	120.4	C112—C111—C110	120.0
C58—C59—O11	118.50 (14)	C112—C111—D111	120.0
C58—C59—C54	122.03 (15)	C110—C111—D111	120.0
O11—C59—C54	119.22 (15)	C113—C112—C111	120.0
C61—C60—C65	121.49 (14)	C113—C112—D112	120.0
C61—C60—O12	119.68 (14)	C111—C112—D112	120.0
C65—C60—O12	118.65 (14)	C112—C113—C108	120.0
C62—C61—C60	116.82 (15)	C112—C113—D113	120.0
C62—C61—C72	119.58 (15)	C108—C113—D113	120.0
C60—C61—C72	123.55 (15)	C108—C114—D11A	109.5
C63—C62—C61	121.98 (15)	C108—C114—D11B	109.5
C63—C62—H62	119.0	D11A—C114—D11B	109.5
C61—C62—H62	119.0	C108—C114—D11C	109.5
O16—C63—C62	124.31 (15)	D11A—C114—D11C	109.5
O16—C63—C64	115.61 (15)	D11B—C114—D11C	109.5
C62—C63—C64	120.08 (15)	C116—C115—C120	120.0
C63—C64—C65	119.73 (15)	C116—C115—C121	119.7 (4)
C63—C64—H64	120.1	C120—C115—C121	120.3 (4)
C65—C64—H64	120.1	C117—C116—C115	120.0
C64—C65—C60	119.50 (15)	C117—C116—D116	120.0
C64—C65—C66	117.14 (14)	C115—C116—D116	120.0
C60—C65—C66	123.34 (14)	C116—C117—C118	120.0
C67—C66—C71	119.21 (15)	C116—C117—D117	120.0
C67—C66—C65	117.61 (14)	C118—C117—D117	120.0
C71—C66—C65	123.14 (14)	C117—C118—C119	120.0
C68—C67—C66	120.13 (15)	C117—C118—D118	120.0
C68—C67—H67	119.9	C119—C118—D118	120.0
C66—C67—H67	119.9	C120—C119—C118	120.0
O17—C68—C67	115.78 (15)	C120—C119—D119	120.0
O17—C68—C69	124.23 (15)	C118—C119—D119	120.0
C67—C68—C69	119.98 (15)	C119—C120—C115	120.0
C68—C69—C70	121.84 (16)	C119—C120—D120	120.0
C68—C69—H69	119.1	C115—C120—D120	120.0
C70—C69—H69	119.1	C115—C121—D12A	109.5
C69—C70—C71	117.07 (15)	C115—C121—D12B	109.5
C69—C70—C78	119.88 (16)	D12A—C121—D12B	109.5
C71—C70—C78	123.04 (16)	C115—C121—D12C	109.5
O13—C71—C70	117.83 (14)	D12A—C121—D12C	109.5
O13—C71—C66	120.66 (14)	D12B—C121—D12C	109.5
			
O1—C1—C2—C3	175.21 (16)	C66—C67—C68—C69	2.6 (2)
C6—C1—C2—C3	1.6 (3)	O17—C68—C69—C70	175.70 (15)
C1—C2—C3—C4	−1.6 (3)	C67—C68—C69—C70	−3.7 (3)
C2—C3—C4—C5	0.7 (3)	C68—C69—C70—C71	−0.5 (2)
C3—C4—C5—C6	0.2 (3)	C68—C69—C70—C78	178.19 (16)
C2—C1—C6—C5	−0.7 (3)	C69—C70—C71—O13	−178.17 (14)
O1—C1—C6—C5	−174.08 (15)	C78—C70—C71—O13	3.2 (2)
C2—C1—C6—C7	−178.26 (16)	C69—C70—C71—C66	5.7 (2)
O1—C1—C6—C7	8.3 (2)	C78—C70—C71—C66	−172.86 (16)
C4—C5—C6—C1	−0.2 (3)	C67—C66—C71—O13	177.16 (14)
C4—C5—C6—C7	177.48 (18)	C65—C66—C71—O13	−0.2 (2)
C1—C6—C7—C12	42.9 (2)	C67—C66—C71—C70	−6.9 (2)
C5—C6—C7—C12	−134.62 (17)	C65—C66—C71—C70	175.81 (15)
C1—C6—C7—C8	−143.71 (17)	C62—C61—C72—C75	−117.75 (18)
C5—C6—C7—C8	38.7 (2)	C60—C61—C72—C75	59.5 (2)
C12—C7—C8—C9	1.9 (2)	C62—C61—C72—C74	1.1 (2)
C6—C7—C8—C9	−171.71 (16)	C60—C61—C72—C74	178.41 (18)
C7—C8—C9—C10	0.8 (3)	C62—C61—C72—C73	120.23 (18)
C8—C9—C10—C11	−2.0 (3)	C60—C61—C72—C73	−62.5 (2)
C9—C10—C11—C12	0.5 (3)	C69—C70—C78—C79	114.9 (2)
C10—C11—C12—O2	176.97 (14)	C71—C70—C78—C79	−66.5 (2)
C10—C11—C12—C7	2.4 (2)	C69—C70—C78—C81	−122.57 (18)
C8—C7—C12—C11	−3.5 (2)	C71—C70—C78—C81	56.0 (2)
C6—C7—C12—C11	170.10 (15)	C69—C70—C78—C80	−4.0 (2)
C8—C7—C12—O2	−178.05 (14)	C71—C70—C78—C80	174.55 (17)
C6—C7—C12—O2	−4.5 (2)	C87—C82—C83—C84	0.6 (3)
C18—C13—C14—C15	4.6 (2)	O14—C82—C83—C84	−174.73 (17)
O3—C13—C14—C15	179.17 (15)	C82—C83—C84—C85	−0.9 (3)
C18—C13—C14—C25	−172.95 (15)	C83—C84—C85—C86	0.1 (3)
O3—C13—C14—C25	1.7 (2)	C84—C85—C86—C87	0.9 (3)
C13—C14—C15—C16	−2.3 (3)	C85—C86—C87—C82	−1.1 (3)
C25—C14—C15—C16	175.28 (17)	C85—C86—C87—C88	175.52 (19)
C14—C15—C16—C17	−1.0 (3)	C83—C82—C87—C86	0.4 (3)
C14—C15—C16—O7	−179.87 (17)	O14—C82—C87—C86	175.67 (16)
O7—C16—C17—C18	−179.04 (17)	C83—C82—C87—C88	−176.36 (17)
C15—C16—C17—C18	2.2 (3)	O14—C82—C87—C88	−1.1 (2)
O3—C13—C18—C17	−177.95 (14)	C86—C87—C88—C93	138.6 (2)
C14—C13—C18—C17	−3.5 (2)	C82—C87—C88—C93	−44.8 (3)
O3—C13—C18—C19	−3.3 (2)	C86—C87—C88—C89	−44.0 (3)
C14—C13—C18—C19	171.24 (15)	C82—C87—C88—C89	132.6 (2)
C16—C17—C18—C13	0.0 (2)	C93—C88—C89—C90	2.7 (3)
C16—C17—C18—C19	−175.17 (15)	C87—C88—C89—C90	−174.8 (2)
C13—C18—C19—C20	−57.3 (2)	C88—C89—C90—C91	−0.7 (4)
C17—C18—C19—C20	117.56 (16)	C89—C90—C91—C92	−1.6 (4)
C13—C18—C19—C24	130.59 (17)	C90—C91—C92—C93	1.8 (3)
C17—C18—C19—C24	−54.5 (2)	C91—C92—C93—C88	0.2 (3)
C24—C19—C20—C21	1.1 (2)	C91—C92—C93—O15	−177.48 (19)
C18—C19—C20—C21	−171.10 (14)	C89—C88—C93—C92	−2.4 (3)
C19—C20—C21—O8	−177.46 (15)	C87—C88—C93—C92	175.04 (18)
C19—C20—C21—C22	4.9 (2)	C89—C88—C93—O15	175.30 (17)
O8—C21—C22—C23	176.80 (15)	C87—C88—C93—O15	−7.2 (3)
C20—C21—C22—C23	−5.4 (3)	C2—C1—O1—P1	121.05 (15)
C21—C22—C23—C24	−0.3 (2)	C6—C1—O1—P1	−65.25 (19)
C21—C22—C23—C31	177.82 (15)	C11—C12—O2—P1	110.45 (14)
C20—C19—C24—O4	173.79 (13)	C7—C12—O2—P1	−74.80 (16)
C18—C19—C24—O4	−14.1 (2)	C18—C13—O3—P1	−56.55 (19)
C20—C19—C24—C23	−7.0 (2)	C14—C13—O3—P1	128.80 (14)
C18—C19—C24—C23	165.06 (14)	C19—C24—O4—P2	−62.22 (17)
C22—C23—C24—C19	6.5 (2)	C23—C24—O4—P2	118.58 (13)
C31—C23—C24—C19	−171.59 (14)	C36—C35—O5—P2	103.54 (14)
C22—C23—C24—O4	−174.29 (14)	C40—C35—O5—P2	−78.83 (15)
C31—C23—C24—O4	7.6 (2)	C45—C46—O6—P2	120.59 (14)
C15—C14—C25—C27	114.46 (18)	C41—C46—O6—P2	−65.06 (19)
C13—C14—C25—C27	−68.1 (2)	C17—C16—O7—C29	−6.2 (3)
C15—C14—C25—C28	−122.95 (18)	C15—C16—O7—C29	172.64 (18)
C13—C14—C25—C28	54.5 (2)	C20—C21—O8—C30	1.4 (2)
C15—C14—C25—C26	−4.7 (2)	C22—C21—O8—C30	179.10 (15)
C13—C14—C25—C26	172.79 (18)	C49—C48—O10—P3	117.57 (14)
C22—C23—C31—C32	109.21 (17)	C53—C48—O10—P3	−69.48 (19)
C24—C23—C31—C32	−72.75 (19)	C58—C59—O11—P3	109.54 (15)
C22—C23—C31—C33	−10.5 (2)	C54—C59—O11—P3	−76.13 (17)
C24—C23—C31—C33	167.54 (15)	C61—C60—O12—P3	113.49 (14)
C22—C23—C31—C34	−128.68 (16)	C65—C60—O12—P3	−71.29 (17)
C24—C23—C31—C34	49.3 (2)	C70—C71—O13—P4	140.53 (13)
O5—C35—C36—C37	179.84 (14)	C66—C71—O13—P4	−43.4 (2)
C40—C35—C36—C37	2.3 (2)	C83—C82—O14—P4	−111.33 (17)
C35—C36—C37—C38	−0.7 (2)	C87—C82—O14—P4	73.18 (19)
C36—C37—C38—C39	−1.3 (3)	C92—C93—O15—P4	−99.77 (18)
C37—C38—C39—C40	1.8 (3)	C88—C93—O15—P4	82.43 (19)
C36—C35—C40—C39	−1.8 (2)	C62—C63—O16—C76	2.1 (3)
O5—C35—C40—C39	−179.30 (14)	C64—C63—O16—C76	−176.72 (17)
C36—C35—C40—C41	175.77 (15)	C67—C68—O17—C77	−161.75 (16)
O5—C35—C40—C41	−1.8 (2)	C69—C68—O17—C77	18.8 (2)
C38—C39—C40—C35	−0.3 (2)	C1—O1—P1—O3	−69.81 (13)
C38—C39—C40—C41	−177.82 (15)	C1—O1—P1—O2	25.75 (13)
C35—C40—C41—C46	42.8 (2)	C1—O1—P1—Rh1	159.97 (11)
C39—C40—C41—C46	−139.72 (17)	C13—O3—P1—O1	−134.97 (12)
C35—C40—C41—C42	−134.81 (17)	C13—O3—P1—O2	124.39 (13)
C39—C40—C41—C42	42.6 (2)	C13—O3—P1—Rh1	−9.31 (14)
C46—C41—C42—C43	−1.1 (3)	C12—O2—P1—O1	61.61 (12)
C40—C41—C42—C43	176.69 (16)	C12—O2—P1—O3	162.76 (11)
C41—C42—C43—C44	0.9 (3)	C12—O2—P1—Rh1	−65.95 (12)
C42—C43—C44—C45	0.2 (3)	C46—O6—P2—O5	23.49 (13)
C43—C44—C45—C46	−1.1 (3)	C46—O6—P2—O4	−74.13 (13)
C44—C45—C46—C41	1.0 (3)	C46—O6—P2—Rh1	154.98 (11)
C44—C45—C46—O6	175.23 (15)	C35—O5—P2—O6	64.68 (11)
C42—C41—C46—C45	0.1 (2)	C35—O5—P2—O4	166.79 (10)
C40—C41—C46—C45	−177.57 (15)	C35—O5—P2—Rh1	−60.82 (11)
C42—C41—C46—O6	−173.99 (14)	C24—O4—P2—O6	−134.54 (11)
C40—C41—C46—O6	8.3 (2)	C24—O4—P2—O5	124.21 (11)
C53—C48—C49—C50	0.0 (3)	C24—O4—P2—Rh1	−8.87 (12)
O10—C48—C49—C50	172.80 (15)	C48—O10—P3—O12	−61.69 (13)
C48—C49—C50—C51	0.4 (3)	C48—O10—P3—O11	35.82 (14)
C49—C50—C51—C52	−0.1 (3)	C48—O10—P3—Rh1	163.45 (11)
C50—C51—C52—C53	−0.7 (3)	C60—O12—P3—O10	−145.21 (12)
C49—C48—C53—C52	−0.7 (3)	C60—O12—P3—O11	113.45 (12)
O10—C48—C53—C52	−173.36 (15)	C60—O12—P3—Rh1	−21.42 (14)
C49—C48—C53—C54	177.79 (16)	C59—O11—P3—O10	53.92 (12)
O10—C48—C53—C54	5.2 (2)	C59—O11—P3—O12	154.33 (12)
C51—C52—C53—C48	1.1 (3)	C59—O11—P3—Rh1	−66.72 (12)
C51—C52—C53—C54	−177.48 (18)	C82—O14—P4—O13	56.31 (14)
C48—C53—C54—C55	−140.80 (18)	C82—O14—P4—O15	−41.83 (14)
C52—C53—C54—C55	37.7 (3)	C71—O13—P4—O14	156.89 (13)
C48—C53—C54—C59	42.1 (2)	C71—O13—P4—O15	−103.00 (13)
C52—C53—C54—C59	−139.37 (18)	C93—O15—P4—O14	−51.64 (14)
C59—C54—C55—C56	0.8 (3)	C93—O15—P4—O13	−150.51 (13)
C53—C54—C55—C56	−176.33 (17)	C99—C94—C95—C96	0.0
C54—C55—C56—C57	0.3 (3)	C100—C94—C95—C96	−178.8 (2)
C55—C56—C57—C58	−0.8 (3)	C94—C95—C96—C97	0.0
C56—C57—C58—C59	0.2 (3)	C95—C96—C97—C98	0.0
C57—C58—C59—O11	175.17 (15)	C96—C97—C98—C99	0.0
C57—C58—C59—C54	1.0 (3)	C97—C98—C99—C94	0.0
C55—C54—C59—C58	−1.5 (2)	C95—C94—C99—C98	0.0
C53—C54—C59—C58	175.65 (16)	C100—C94—C99—C98	178.8 (2)
C55—C54—C59—O11	−175.62 (15)	C106—C101—C102—C103	0.0
C53—C54—C59—O11	1.5 (2)	C107—C101—C102—C103	−179.3 (6)
C65—C60—C61—C62	5.2 (2)	C101—C102—C103—C104	0.0
O12—C60—C61—C62	−179.67 (14)	C102—C103—C104—C105	0.0
C65—C60—C61—C72	−172.12 (15)	C103—C104—C105—C106	0.0
O12—C60—C61—C72	3.0 (2)	C104—C105—C106—C101	0.0
C60—C61—C62—C63	0.4 (2)	C102—C101—C106—C105	0.0
C72—C61—C62—C63	177.87 (16)	C107—C101—C106—C105	179.3 (5)
C61—C62—C63—O16	177.17 (16)	C113—C108—C109—C110	0.0
C61—C62—C63—C64	−4.1 (3)	C114—C108—C109—C110	178.6 (8)
O16—C63—C64—C65	−179.00 (14)	C108—C109—C110—C111	0.0
C62—C63—C64—C65	2.2 (2)	C109—C110—C111—C112	0.0
C63—C64—C65—C60	3.3 (2)	C110—C111—C112—C113	0.0
C63—C64—C65—C66	−175.42 (14)	C111—C112—C113—C108	0.0
C61—C60—C65—C64	−7.2 (2)	C109—C108—C113—C112	0.0
O12—C60—C65—C64	177.68 (13)	C114—C108—C113—C112	−178.6 (9)
C61—C60—C65—C66	171.49 (14)	C120—C115—C116—C117	0.0
O12—C60—C65—C66	−3.6 (2)	C121—C115—C116—C117	179.7 (6)
C64—C65—C66—C67	−60.49 (19)	C115—C116—C117—C118	0.0
C60—C65—C66—C67	120.80 (17)	C116—C117—C118—C119	0.0
C64—C65—C66—C71	116.88 (17)	C117—C118—C119—C120	0.0
C60—C65—C66—C71	−61.8 (2)	C118—C119—C120—C115	0.0
C71—C66—C67—C68	2.6 (2)	C116—C115—C120—C119	0.0
C65—C66—C67—C68	−179.96 (14)	C121—C115—C120—C119	−179.7 (6)
C66—C67—C68—O17	−176.85 (14)		
